# Advocating for automation in animal research: using home cage monitoring to advance welfare, reproducibility and scientific openness

**DOI:** 10.1242/dmm.052669

**Published:** 2025-10-29

**Authors:** COST Action TEATIME CA20135 welfare group

**Affiliations:** https://www.cost.eu/actions/CA20135/

## Abstract

**Summary:** Members of the COST Action TEATIME CA20135 welfare group advocate for automation in animal research to help realise the promise of laboratory animal science.

Recent advances in artificial intelligence (AI) and machine learning (ML) technologies present significant opportunities to drive substantial progress in laboratory animal science. However, to realise this potential, the animal research community must radically change the way in which it generates, annotates and shares large datasets to enable and foster the required interdisciplinary collaboration.

Herein, we highlight the importance that new developments in automated welfare and phenotypic analysis for laboratory animals offer in reducing subjectivity, limiting influencing factors, increasing the rigor, robustness and quality of data, and enhancing the relevance and translatability of animal studies. In particular, we discuss the opportunities and potential impact of home cage monitoring (HCM) systems on reducing both the numbers of animals used in research (reduction) and their discomfort (refinement). We also address the significant logistical, cultural and resource challenges that the research animal community must overcome to attract and collaborate effectively with informaticians and data scientists to realise these ambitions.

## Home cage monitoring

**Fig. 1.**
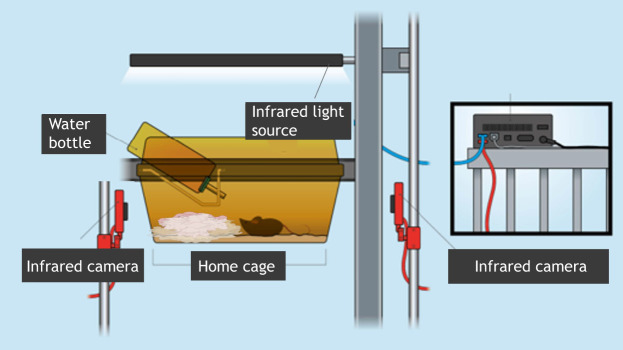
An example of a home cage monitoring (HCM) setup, including lighting and infrared cameras mounted on typical cage racking.

Since 2021, the COST TEATIME Action consortium has brought together animal research professionals from across Europe to discuss the development of novel and emerging technologies that enable the 24/7 collection of data from animals in their home cages. The home cage context is currently defined as a system that collects data on animals housed in enclosures that provide food, water, social interaction and protection from external threats within an enriched environment. A vast range of HCM systems, mostly for mice, have been catalogued. Equipment ranges from sophisticated cages with integrated monitoring and data-processing software, commercially available from various companies, to systems assembled from readily available and affordable components, such as video cameras paired with low-cost commodity computers (Fig. 1).

The greatest advantage of these systems is that they allow the monitoring of animals 24/7, in a non-intrusive way over extended time periods. This enables the detection of early, subtle or sporadic indicators of disease progression, outcomes of experimental interventions or welfare concerns. Such effects are frequently missed in the routine out-of-cage behavioural testing or brief daily observations of nocturnal animals, especially rodents, which are typically carried out during daylight hours and may fail to detect changes over time. Depending on the systems and equipment used, parameters can be measured directly – for example, measurements of activity levels and the emission of (ultra)sonic vocalisations (Wöhr and Schwarting, 2013; Premoli et al., 2023) – or can be derived analytically from collections of measurements, such as in the analysis of circadian rhythms, sleep patterns and social behaviours. These parameters can be combined with physiological measures – for example, heart rate and body temperature – tailored to specific research questions and/or to assess the welfare and experiences of individual animals.

The scientific and welfare advantages of these systems include the potential to train predictive AI/ML models to recognise more granular disease signatures in animal behaviour, a so-called digital biomarker, thereby allowing immediate warning signals as a basis for early intervention and early humane endpoints. At present, different systems measure distinct parameter sets, and the potential to obtain more power by combining the datasets for meta-analysis has yet to be explored. However, such integration could form the foundation for greater inference in animal studies, whereby a subset of parameters could be used confidently to infer a more robust interpretation of the data.

There are also non-rodent initiatives using AI-based analysis, such as the NHPig project, which aims to ethically replace non-human primates with mini-/micro-pigs in drug development research by developing intelligent animal housing with biosensors for automated data collection. This approach will mirror the state-of-the-art in rodent HCM by essentially scaling these advanced monitoring principles to a larger-animal model.

## The logistical challenge

The extent to which sensors such as microphones and cameras can assess the physiological and behavioural states of animals remains to be fully exploited as ever more sensitive detection systems are developed. Moreover, many welfare definitions are based upon allowing animals to express normal species-specific behaviours. But to do this, we need to define what these are first, and whether they are occurring under husbandry or captivity conditions.

A recent systematic review of HCM (Kahnau et al., 2023) has shown a steady increase in its use. The main behavioural parameters measured were found to be locomotor activity, feeding and social behaviours, whereas the main physiological parameters were heart rate and electrocardiogram. Changes in the external appearance of an animal are rarely examined in the home cages, probably due to the complexity of automation and the limits of resolution in the assessment of such complex features.

To be exploited to its full potential as a tool for monitoring animal wellbeing, HCM systems must strive to provide data in real time. Ideally, a system must detect when an animal's behavioural or physiological parameters deviate from normal or set ranges, and immediately notify the animal carer that the animal may require attention. The integration of multiple measurements to reflect the multidimensional nature of the animal at any given time point would be the ultimate goal and has begun for some parameters (Talbot et al., 2022). Of course, this requires that normal ranges are well enough understood to set such a range and poses issues relating to sensitivity and specificity to limit false negative and positive notifications. Similarly, the ability to rapidly analyse and compare complex datasets across multiple days would allow the monitoring of disease progression, the effects of scientific procedures and the impact of therapeutic interventions.

In addition, the sporadic occurrence of disease phenotypes, such as certain types of seizures, or welfare indicators that are not displayed constantly makes it challenging to capture enough data to train automation systems. Integrating expert scientific and welfare knowledge back into HCM systems to identify these types of behaviours and further inform real-time monitoring is also an unaddressed challenge. Humans and machines training each other to get better at welfare assessment is indeed an exciting prospect. Moreover, extending the analysis to multiple animals that are housed in groups and are indistinguishable from each other adds an additional layer of complexity to the ability to identify a specific individual with compromised wellbeing within the group. However, recording these types of complex behaviours will ultimately deliver the opportunity of alignment of animal data with human movement being recorded for medical diagnosis.

## Barriers to uptake

Discussion around the advantages of being able to constantly monitor experimental animals throughout an experiment, or indeed their entire lifetimes, has been ongoing for many years. As has the reluctance of the animal research community to embrace these technologies due to issues with scalability and the lack of cross-disciplinary collaboration between informaticians, engineers and biomedical scientists. Although many different systems exist, these are largely used for specialist requirements, typically at small scale within large, well-funded institutes. However, there are many researchers who are keen to adopt these technologies and who are looking towards technologies that are more flexible or have a lower cost of entry.

Putting aside the requirement for the resources to purchase and maintain equipment, the ability to manage, store and analyse the potentially vast amounts of data of differing types that result from HCM is far outside the remit of most animal facilities. Storage and data processing alone are costly and have environmental and energy implications. Understandably, research groups are often focused on individual areas of scientific interest and will develop software tools that are only applicable to that interest. Commercially available equipment, with proprietary analysis software tools, provides a partial solution for some researchers; however, many may require further transparent validation of these tools, raw data quality assessment/control, data processing and analytic power determination, before integrating them into experimental and/or welfare pipelines. In addition, the high technical barrier, which may include the need to understand complex coding and algorithms, or the ability to download raw data of various types for analysis and comparison, limit the broader interrogation of these systems.

## Tackling the problem

In this era of increasingly more sophisticated analysis of human healthcare data, HCM has the potential to revolutionise preclinical and discovery animal research through the way in which large biobank datasets are used to influence human health (e.g. Coppola et al., 2019). Progress in AI and ML across many scientific fields has only been possible because of the availability of large, openly accessible datasets used to train, refine and validate computer algorithms. It is plausible that similarly large and complex datasets – such as animal movement data, cage images and audio recordings from HCM – could collectively be of interest and usable by computer scientists as well as biologists. However, several substantial changes would need to be initiated, embraced and embedded into the culture of this community to realise the dream of sensitive, informative phenotyping data concurrent with exemplary animal care.

### Encouraging and attracting collaboration from the computer science community

Few individual researchers or laboratories will ever have the combined skill set to recognise and interpret animal behaviour and simultaneously develop computer code to automate real-time data analysis. The ability to specify biological data requirements and to develop the algorithms and methods to digitally deliver these data typically requires the collaboration of multidisciplinary teams from the outset of a project, with ongoing expertise provided throughout its duration. For early-career computer/data scientists, without biological insight, working within animal research might not be an attractive proposition unless we can produce easily accessible, rich data sources, addressing interesting scientific problems, and conceptualise the value of projects appropriately. In particular, the availability of substantial expert-annotated datasets that use well-defined and published definitions is essential to garner the interest required. Indeed, so called ‘benchmarks’ have fuelled much of ML research in the past decade (e.g. Mathis et al., 2020).

Behaviour experimentation and AI/ML are skill sets that are both vital to HCM innovation, but rarely are collaborators experts in both. To engage AI/ML engineers to tackle HCM analysis challenges in depth, the field must first define the biological problems and establish a clear pathway for delivering and benchmarking solutions. ML engineers often work within the emerging paradigm of ML operations, which incorporate user feedback (in this case, from the HCM field) to assess product fit through iterative development. This approach also requires data quality checks before any work begins on low-quality data and needs to ensure that data are accessible, trustworthy and valuable on their own merit; value or new features are added iteratively to a stable data product. We should aim to make available expertly annotated datasets from the outset, using well-defined and published definitions. This task is challenging, especially as annotation is a fundamentally expensive, time and resource-consuming overhead. Those managing these data should engage early and continuously with ML engineers to understand how to shape product development and encourage productive collaboration and even competition.

### Unified data formats/metadata

To share HCM data with non-biological specialists and to attract the skills of expert data scientists, it is imperative that data are accessible and understandable. A requirement to reformat poorly indexed data prior to analysis is unlikely to engage informaticians. Collecting new data in a consistent way and in formats that can be easily combined and merged is essential for building comprehensive data libraries from multiple sources. Increased standardisation of data recording between systems and research groups would greatly facilitate this but is a difficult goal to achieve with the involvement of multiple agencies (suppliers, researcher and facilities). Metadata and version control (to account for changes in over time) are key to the interpretation and sharing of data and, in the case of HCM, this should include the definition of a minimal metadata set (Moresis et al., 2024). All datasets should be collected in compliance with FAIR principles ‘to improve the findability, accessibility, interoperability and reuse of digital assets’.

HCM data are granular, dynamic, multi-dimensional and valuable in terms of monitoring animal welfare. By its analysis, we should aim to quantify complex behavioural organisation, such as social hierarchy or sporadic signs of degenerative diseases. The ability to work with HCM data as a non-expert requires information that can be captured in metadata organised according to their complexity. These data structures are ‘machine readable’ and can be essential to parsing data at scale in large ML applications. Contextual metadata are also key to data interoperability between systems as mapping similar biological data together is essential for the integration of large datasets prior to the training of new ML models. Taking this approach reduces the need for fixed unified data formats. The FAIR principles complement management of a data product, ultimately making the data more useful to non-experts. They do not need to be applied exhaustively early in collaboration, but if aiming to reduce animal use through data reuse, they are recommended as best practice.

### Data management

The collection and storage of data inevitably takes more than just formatting; laboratory information systems for animal facilities are usually developed to collect the details of animal breeding and care. Collecting, moving and storing terabytes of data requires a management strategy that is both robust, to ensure that recording is continuous, and economic, such that only unique datasets or those of scientific value are stored. Multiple data management strategies are available, depending on the specific use case within the community. On-premises-managed resources at a single site are becoming increasingly rare for larger collaborations, as the volume and speed with which datasets are being produced continue to increase. They are constrained by the cost of hardware setup, the availability of IT expertise and the vulnerability to cyberattacks, all of which complicate open data sharing and continuous availability. Federated data management strategies still use on-premises hardware, but distribute data across multiple sites, thereby sharing the cost and management burdens. These are popular strategies in large academic projects, but still require IT and software development expertise to devise a stable federated architecture. Cloud-based data strategies are increasingly popular; however, the current high price of using cloud storage at scale will be restrictive to many.

### Creating reference datasets and open data

Modern methods in AI and ML rely on large amounts of annotated data for training and evaluation/validation. The analysis of that data for correlations and patterns will then be used to make predictions about future states. The datasets delivered by HCM users must therefore be discoverable, user friendly and secure. Costs, storage space and file-size limitations may be barriers to using some common data repositories, which have limits on the amount of data that can be shared without cost (typically 20–50 GB per dataset). Large data types, such as video and neurophysiology recordings, are typically into the multiple terabytes or even petabytes and are even more challenging to store and make available.

Research funders and journals increasingly expect that data published in their journals are made freely available. This is exemplified by the National Institutes of Health (NIH)-endorsed common data elements (an NIH funding requirement for the collection of interoperable metadata). Long-term preservation of data for individual laboratories is also a significant challenge. It would therefore be a logical step for the HCM community to explore the creation of a new central repository for HCM data or the repurposing and exploitation of an existing resource, facilitating the development of methods for new scientific approaches. This would enable the provision of reference datasets for use by research biologists, technology specialists, data scientists and data integrators. A small number of high-quality well-annotated datasets would be an excellent starting point!

Facilitating easy data upload for researchers would be likely to enhance uptake, but could risk that much of the data may not be in the correct format or may lack the necessary metadata, thereby compromising usability. However, more stringent upload requirements may discourage data submission. In addition, the animal research community has issues of security and privacy, which are usually managed at the institutional level and which would require much discussion.

We also need to recognise that the value and need for these reference datasets will evolve. Right now, the field needs access to high-quality raw data and the associated annotations. As methods for primary analysis (essentially feature detection) evolve, it is highly likely that we will need fewer of these bulky datatypes and will be able to focus more on storing vectorised data (e.g. extracted positions and body postures, key points, waveforms) that are much less space hungry and easier to share. However, it's important to only compress data once we have figured out what is essential to make the relevant welfare decisions.

## Call to action!

In conclusion, we implore the following stakeholders to consider the following points:

(1) Funding bodies
  - Consider the value of large collections of data in the context of ‘big data’ experiments and invest in infrastructure, or provide access to existing infrastructure, to support the deposition of HCM data.
  - When funding HCM in grants, consider how the data will be made available to the wider community in a useful format that will attract computer scientists, making this a prerequisite of this type of funding.
(2) Animal researchers
  - Be clear about what you are trying to measure and how you will know you have measured it successfully.
  - Prior to data collection, establish a comprehensive set of metadata and a unified data format that will be followed throughout the experiment.
  - Be prepared to use your expertise to annotate data and share this annotation with others. Do not underestimate the resource required to do this. Equally do not undervalue the upside of doing so.
  - Work with your institutions to adopt a spirit of openness and collaboration, enabling greater data sharing.
  - Support access and training for changing paradigms in animal monitoring.
(3) Data scientists
  - Clarify your needs and requirements in terms of unified datasets.
  - Build semi-automatic annotation tools to get the most out of expert inputs.
  - Engage with the community to enable these exciting developments to become reality.

## Final thoughts

**Fig. 2.**
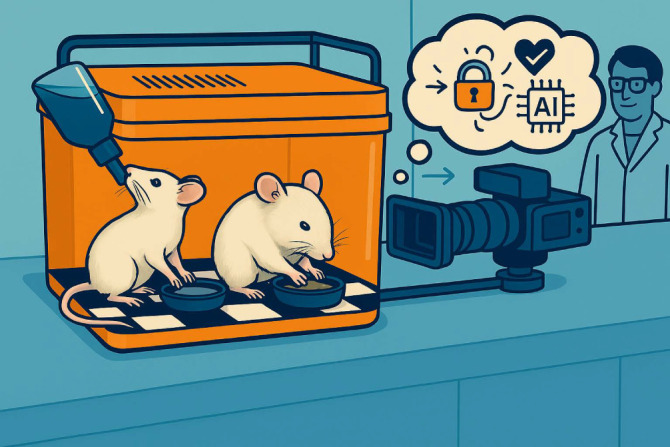
Depicting multiple opportunities for HCM systems in the future in collaboration with AI and computer science.

If we continue working in isolated silos – protecting data and analysis – we risk missing out on major scientific and welfare advancements. But if we invest in collaboration, openness and scalable data infrastructure, we can revolutionise animal research and care. Let's not waste this opportunity (Fig. 2).
